# TolC facilitates the intracellular survival and immunomodulation of *Salmonella* Typhi in human host cells

**DOI:** 10.1080/21505594.2024.2395831

**Published:** 2024-08-26

**Authors:** Ashraf Hussain, Eugene Boon Beng Ong, Prabha Balaram, Asma Ismail, Phua Kia Kien

**Affiliations:** a Institute for Research in Molecular Medicine (INFORMM), Universiti Sains Malaysia, Penang, Malaysia; b John P. Hussman Institute for Human Genomics, University of Miami Miller School of Medicine, Miami, FL USA

**Keywords:** *Salmonella* Typhi, multidrug efflux pump AcrAB-TolC, invasion of bacteria into host cells, mutant induce cytotoxic proinflammatory responses in host cell, immunomodulation of macrophages and epithelial cells, immunomodulatory ability of pathogens

## Abstract

*Salmonella enterica* serovar Typhi (*S*. Typhi) causes typhoid fever, a systemic infection that affects millions of people worldwide. *S*. Typhi can invade and survive within host cells, such as intestinal epithelial cells and macrophages, by modulating their immune responses. However, the immunomodulatory capability of *S*. Typhi in relation to TolC-facilitated efflux pump function remains unclear. The role of TolC, an outer membrane protein that facilitates efflux pump function, in the invasion and immunomodulation of *S*. Typhi, was studied in human intestinal epithelial cells and macrophages. The *tolC* deletion mutant of *S*. Typhi was compared with the wild-type and its complemented strain in terms of their ability to invade epithelial cells, survive and induce cytotoxicity in macrophages, and elicit proinflammatory cytokine production in macrophages. The *tolC* mutant, which has a defective outer membrane, was impaired in invading epithelial cells compared to the wild-type strain, but the intracellular presence of the *tolC* mutant exhibited greater cytotoxicity and induced higher levels of proinflammatory cytokines (*IL-1β* and *IL-8*) in macrophages compared to the wild-type strain. These effects were reversed by complementing the *tolC* mutant with a functional *tolC* gene. Our results suggest that TolC plays a role in *S*. Typhi to efficiently invade epithelial cells and suppress host immune responses during infection. TolC may be a potential target for the development of novel therapeutics against typhoid fever.

## GRAPHICAL ABSTRACT

**Figure:**
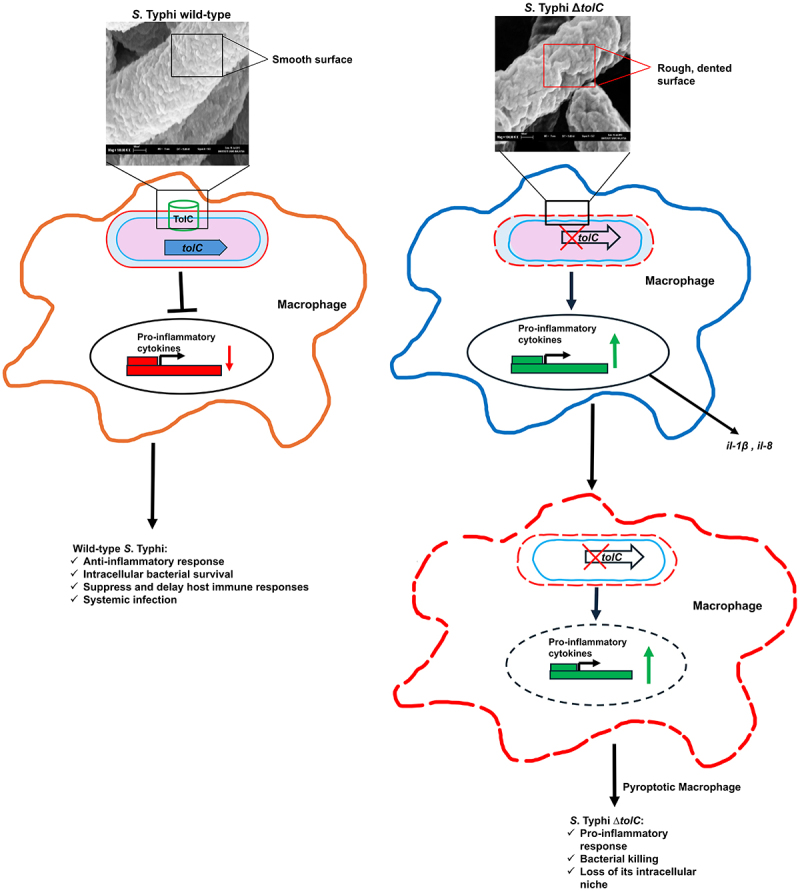

## Introduction

*Salmonella enterica* serovar Typhi (*S*. Typhi), a human-restricted pathogen that causes typhoid fever, can evade and suppress the host’s innate immunity; therefore, its infection does not trigger effective proinflammatory responses [1–3]. Furthermore, *S*. Typhi can inhibit the host’s programmed cell death mechanisms in macrophages [4]. These mechanisms involve caspase-1 and caspase-11-mediated pyroptosis, which produces proinflammatory cytokines in response to cytosolic flagellin and lipopolysaccharides (LPS) from intracellular *Salmonella* [5,6].

AcrAB-TolC is the major multidrug resistance efflux system found in *Escherichia coli* and *Enterobacteriaceae*, including *Salmonella*, and it contributes to the pathogenesis and virulence of several bacterial pathogens [7–13]. TolC, an efflux pump protein, plays a role in pathogen virulence and has been shown to be required for host-suppressive actions [14–18]. A *tolC* mutant of *Francisella tularensis* induces hypercytotoxicity in host cells and triggers an increased production of proinflammatory chemokines in human macrophages compared with the response of the wild-type strain [14]. Defects in bacterial structural integrity can also induce hypercytotoxicity in host cells [19–23]. However, it remains unclear how the removal of *tolC* impacts the structural integrity of *S*. Typhi cells, and whether any resulting structural integrity defects are related to the induction of cell cytotoxicity in the host cell. Therefore, it is suggested that *S*. Typhi has specific virulence factor(s) or mechanisms that are associated with the outer membrane of bacterial cells, which allow it to suppress the innate immune response in the intestinal mucosa, facilitating its systemic dissemination and cytotoxicity [2,3,24]. However, the precise molecular mechanisms and function of outer membrane protein, TolC, in the immunomodulatory ability of *S*. Typhi have yet to be studied.

TolC either secretes the effector, prevents the secretion of the effector, or directly interferes with innate immune pathways. This interference can delay the activation of host cell death and reduce effective proinflammatory responses against intracellular pathogens. To assess this hypothesis, the role of TolC was investigated in the immunomodulatory ability of *S*. Typhi in human intestinal epithelial cells and macrophages, which are the primary targets of *S*. Typhi infection. We found that the *tolC* mutant was impaired in invading epithelial cells compared with the wild-type strain, but the intracellular presence of the *tolC* mutant was hypercytotoxic towards human macrophages. The *tolC* mutant also induced higher levels of proinflammatory response in macrophages than the wild-type strain. During the structural analysis of the *tolC* mutant, it was observed that the Lipopolysaccharide (LPS) patches were not detectable or modified on the damaged cell membrane of the *tolC* mutant. These changes on the cell surface could potentially induce hypercytotoxicity in host cells. To our knowledge, this is the first report of *tolC* deletion-related phenotypes expansion to *S*. Typhi, a pathogen known to exclusively cause systemic infections in humans, showing the intracellular presence of the outer membrane defective *tolC* mutant may cause hypercytotoxicity in human macrophages.

## Materials and methods

### Bacterial strains, growth, and construction of the *S*. *Typhi* mutant

The wild-type strain of *Salmonella enterica* serovar Typhi (*S*. Typhi), designated as ST-WT, was isolated from a patient with acute typhoid fever and used in this study. This study adhered to the ethical guidelines of the Declaration of Helsinki and the EEC directive of 1986. The *S*. Typhi strain was isolated at the Hospital Universiti Sains Malaysia (HUSM) and stored in the Institute for Research in Molecular Medicine (INFORMM) Bank, Kubang Kerian, Kelantan, Malaysia. The strain collection and usage received ethical approval from the Universiti Sains Malaysia Human Ethical Committee, located in Kubang Kerian, Malaysia (Ethical clearance number: USMKK/PPP/JEPeM [229.3. (03)]. Informed consent was obtained from all participants prior to their enrolment in the study. The bacterial strains and plasmids were utilized as mentioned in our previous study [10]. All strains were cultured in Luria−Bertani (LB) agar and broth (Hi-media) at 37°C with the addition of appropriate antibiotics for selection.

### Construction of the tolC mutant

The one-step chromosomal gene inactivation method was used [25,26] to construct the *tolC* deletion mutant (ST-Δ*tolC*) by replacing the *tolC* gene of the ST-WT strain with the kanamycin resistance gene *aph* (3’)-II. A complementation mutant (ST-∆*tolC+*) was made by cloning the *tolC* gene with its native promoter into the pKK223-3 plasmid and transforming it into ST-Δ*tolC* [10].

### Invasion assays

The invasion of *S*. Typhi strains was tested using *in vitro* assays with THP-1 macrophages and HT-29 epithelial cells, following the methods of Dibb-Fuller, Allen-Vercoe [27] and Buckley, Webber [28]. The *S*. Typhi strains were cultured overnight in 10 ml of LB broth at 37°C and used to infect the confluent cell monolayers in 6-well plates for 2 h at a multiplicity of infection (MOI) of 50. The invasion assay was used (also called gentamicin protection assay) to measure intracellular bacteria as described previously by Amy, Velge [29]. After 2 h of infection, the cells were washed six times with PBS (pH 7.3) and disrupted with 1 ml cold distilled water (4°C) [30]. Viable intracellular bacteria were counted as CFU/mL after plating serial dilutions with PBS. The mean CFU/mL was calculated for each strain and expressed as a relative percentage of the wild-type values for each replicate. All quantitative invasion assays were performed separately for each strain in triplicate. The overall mean CFU/mL for each strain was calculated. The ST-∆*tolC* and ST-∆*tolC*+ were compared with the ST-WT reference strain using Student’s t-test.

### Reverse transcription PCR of il-1β and il-8 gene expression

Reverse transcription polymerase chain reaction (RT-PCR) was conducted to measure the mRNA expression of *il-1β* and *il-8* target genes in this study. Primers for host response genes were sourced from the primer bank of the Harvard database [31], as described in Table 1. The samples were prepared in the same manner as in the invasion assay, but in the final step, cell lysis was performed using an RNA extraction kit. RNA was extracted from infected macrophages using the Qiagen RNeasy Mini Kit, following the manufacturer’s instructions. Any possible DNA contamination was removed by treatment with DNase I (Sigma). The purity and concentration of RNA were determined by measuring the optical density at 230, 260, and 280 nm using NanoDrop 2000C (Thermo Scientific, USA) prior to use. The 260/280 ratio, which is used to assess the purity of DNA and RNA, was also calculated. A ratio of ~ 1.8 is generally accepted as “pure” for DNA, while a ratio of ~ 2.0 is generally accepted as “pure” for RNA. The 260/230 ratio, used as a secondary measure of nucleic acid purity, ideally should be greater than 2.0. The quality of the RNA was assessed by gel electrophoresis and ethidium bromide staining.

**Table 1.** qPCR primers for macrophage cells.

N0	primer Description	Sequence	Amplicon	Source or reference
1	*il-1β*_F	5-ATGATGGCTTATTACAGTGGCAA-3	132bp	Primer Bank ID #27894305c1
	*il-1β*_R	5-GTCGGAGATTCGTAGCTGGA-3		
2	*il-8*_F	5-ACTGAGAGTGATTGAGAGTGGAC-3	112bp	Primer Bank ID #10834978a2
	*il-8*_R	5-AACCCTCTGCACCCAGTTTTC-3		
3	*GAPDH*_F	5-ACAACTTTGGTATCGTGGAAGG-3	101bp	Primer Bank ID #378404907c2
	*GAPDH*_R	5-GCCATCACGCCACAGTTTC-3		

In this experiment, the gene expression of *IL-1β* and *IL-8* was normalized using *GAPDH* (glyceraldehyde-3-phosphate dehydrogenase) as the reference gene. The macrophages infected with ST-WT, ST-Δ*tolC*, and ST-Δ*tolC*+ were studied, with the ST-WT-infected macrophages serving as the control sample. Initially, 1 µg of DNase-treated total RNA from at least three independent cultures was reverse transcribed using random hexamers and a Superscript III 1st Strand Kit (Invitrogen, Cat #18080-051). Amplification was then performed using the QuantiFast SYBR Green PCR Kit (Qiagen) on an Applied Biosystems™ 7500 Real-Time PCR System, following the manufacturer’s instructions. The Delta Ct (ΔCt) for each sample was calculated by subtracting the Ct value of the *GAPDH* housekeeping gene from the Ct value of the gene of interest (ΔCt = Ct__gene of interest_ - Ct__*GAPDH*_). The double delta Ct (ΔΔCt) was then calculated by subtracting the ΔCt of the control sample (ST-WT-infected THP-1) from the ΔCt of the test samples (THP-1 cells infected with ST-∆*tolC* or THP-1 cells infected with ST-∆*tolC*+), using the formula (ΔΔCt = ΔCt__test sample_ - ΔCt__control sample_). Finally, the relative gene expression was calculated using the formula (Relative expression = 2−^ΔΔCt^). The expression of target the gene is presented as the fold change relative to the THP-1-cell infected with the ST-WT strain [32]. Data were obtained in three separate experiments with three technical replicates. The THP-1 cells infected with ST-∆*tolC* and THP-1 cells infected with ST-∆*tolC+* were compared with ST-WT-infected THP-1 cells as an experimental control using Student’s t-test. *p* values of less than 0.05 were considered significant.

### LDH assay

To measure the cytotoxicity of the infected macrophages, the lactate dehydrogenase (LDH) assay was performed. LDH is an enzyme that is rapidly released into the cell culture medium when the plasma membrane is damaged. The amount of LDH in the medium reflects the degree of cell death. The CytoTox 96(R) Non-Radio Cytotoxicity Assay (Promega, USA) was used according to the manufacturer’s protocols. This assay quantifies LDH activity in the medium by converting a tetrazolium salt into a red formazan product that can be measured spectrophotometrically.

The cells were washed three times with PBS, then fresh RPMI 1640 without antibiotics was added, and the plates were incubated for 4 hours. The conditioned media was collected and analysed for LDH release. The background of LDH release was measured in the medium conditioned by uninfected cells, and the total LDH release (indexed as 100%) was calculated in uninfected cells that were lysed by freezing and thawing. The percentage of LDH release was calculated by subtracting the background LDH release value from the LDH release value of the infected cells, dividing this number by the total LDH release value, and then multiplying it by 100.

(2) $$\begin{array}{rl} & \mathrm{Cytotoxicity}\;\left(\%\right)=[(\mathrm{Treated}\;\mathrm{sample}\;A490_{\mathrm{nm}}\;\\ & -\mathrm{Untreated}\;\mathrm{sample}\;490_{\mathrm{nm}})(\mathrm{Lysed}\;\mathrm{sample}\;\\ & A490_{\mathrm{nm}}-\mathrm{Untreated}\;\mathrm{sample}\;A490_{\mathrm{nm}})]X\;100. \end{array}$$

Treated sample A490_nm_ was the absorbance of the LDH released release from the test sample. Untreated sample A490_nm_ was the absorbance of the spontaneous release of LDH released from the untreated cells (negative control). Lysed sample A490_nm_ was the absorbance of LDH released from the lysed cells (positive control).

### TUNEL staining

To measure the pyroptosis of the infected macrophages, TUNEL staining was performed. TUNEL is a technique that detects DNA fragmentation, a hallmark of programmed cell death. The DeadEnd(TM) Fluorometric TUNEL System (Promega, USA) was used according to the manufacturer’s protocols. This system labels the 3’-OH ends of the fragmented DNA with fluorescein-dUTP, which can be detected by fluorescence microscopy.

The infected macrophages were analysed as described above for TUNEL staining. The cells were visualized using fluorescence microscopy, and the images were captured using a Spot camera. Then, the images were processed using an image analyser and the percentage of TUNEL-positive cells was calculated. Finally, the number of TUNEL-positive cells was divided by the total number of cells in ten different fields.

### Transmission electron microscopy analysis

Transmission electron microscopy (TEM) was used to examine the cytotoxic effect of *S*. Typhi invasion of THP-1 macrophages and HT-29 epithelial cells. These cells were infected with ST-WT, ST-Δ*tolC*, or ST-Δ*tolC+* strains at an MOI of 50 for 2 h. The samples for TEM were prepared according to the invasion assay, except for the cell lysis step. The infected cells were harvested with cold PBS-EDTA (0.1%), washed with PBS, and fixed overnight in PLP buffer (4% paraformaldehyde, 0.01 M periodate, and 0.2 M L-lysine in 0.1 M phosphate buffer, pH 7.4). The samples were rinsed in distilled water, post-fixed in 1% osmium tetroxide for 30 min, dehydrated in ethanol and acetone, and embedded in Epon resin. Thin sections were prepared with an ultramicrotome (Power tome, Boeckeler) and observed under an EFTEM Libra 120 electron microscope (Carl Zeiss, Germany). Representative electron micrographs for each strain’s infection were obtained. At least three infected cultures per strain were visualized and up to ten images per culture were obtained (minimum of thirty images per strain). Each image shows at least one infected host cell. The images were grouped into two categories: (1) infected cells that had nuclei and intact membranes and (2) infected cells that did not have nuclei and had defective cell membranes.

### Scanning electron microscopy (SEM) of bacterial cells

All three strains (i.e. ST-WT, ST-∆*tolC*, and ST-∆*tolC*+) were processed and observed according to Yuen *et al*. (2012). Briefly, the bacterial strains were grown to the exponential phase in LB broth and centrifuged at 2,000×g for 15 min. The bacterial pellets were fixed with a fixing solution (4% formaldehyde w/v) overnight. Then, the samples were centrifuged at 2,000×g for 15 min, dehydrated through a graded ethanol series (20%, 40%, 60%, 80%, 95%, and 100%), and subjected to gold coating. After being dehydrated, samples were coated with gold, the bacterial cells were viewed using a Leo Supra 50 VP field emission scanning electron microscope (Carl-Zeiss SMT, Oberkochen, Germany) equipped with an Oxford NCA 400 energy dispersion X-ray microanalysis system (Oxford Instruments, Bucks, UK).

## Results

### Invasion of *S*. *Typhi*

The function of *tolC* on host cell invasion and its role in suppressing the host’s response was investigated. To test this hypothesis, two distinct cell lines were selected for experimentation: Epithelial cells (HT-29) and macrophages (THP-1). THP-1 cells show phagocytic activity during invasion assays, which allows the entry of ST-Δ*tolC* into the macrophages. On the other hand, HT-29 cells lack phagocytic activity, thus preventing the entry of ST-Δ*tolC* into the cells.

**Figure 1.**
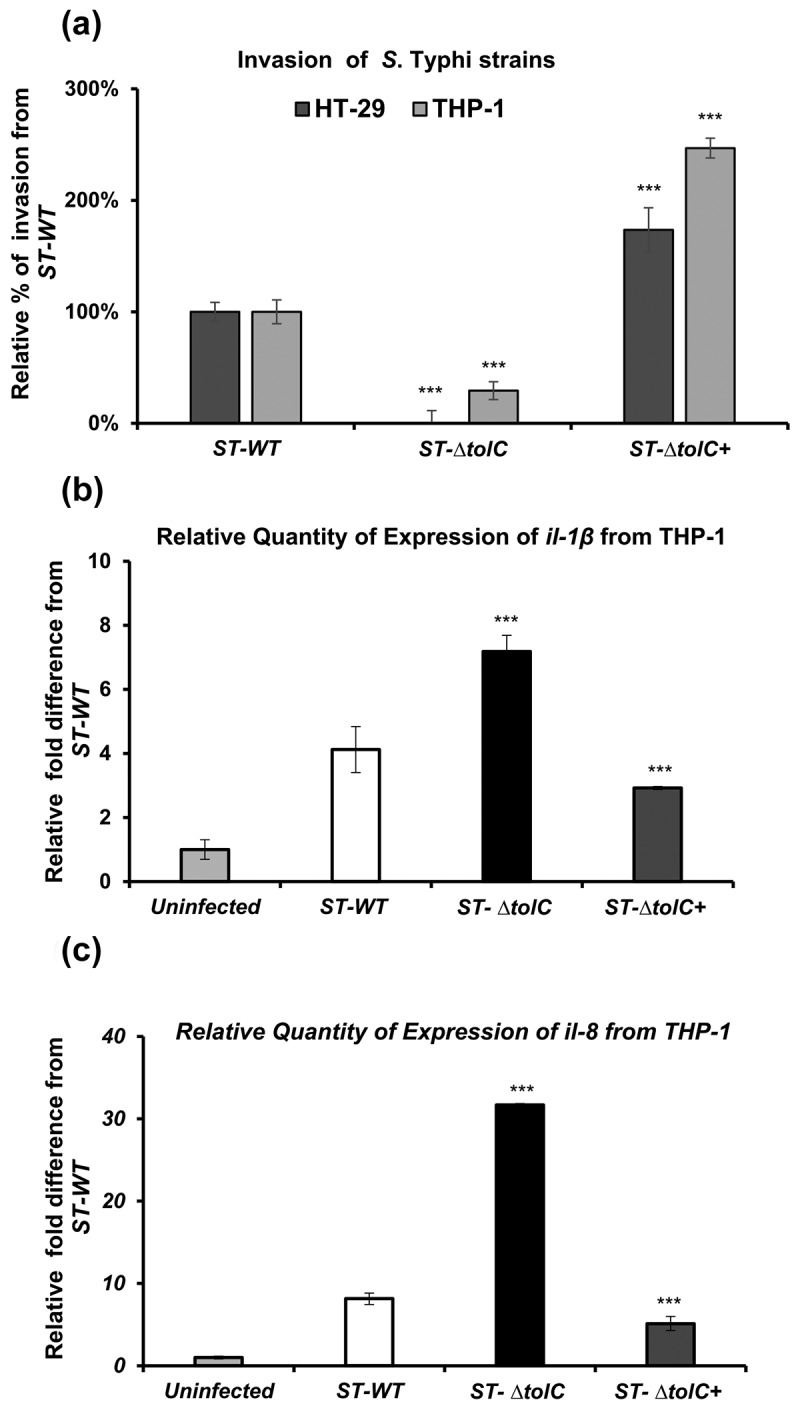
The ST-Δ*tolC* mutant is impaired in invasion and induces proinflammatory chemokines in human macrophages.

The ST-∆*tolC* strain lost the ability to invade host epithelial cells. The ST-∆*tolC* strain invaded significantly less than the ST-WT strain, with its invasion being 0.03% of the ST-WT invasion (***p < 0.001). On the other hand, the ST-∆*tolC+* strain invaded significantly more than the ST-WT strain, with its invasion being 174.00% of the ST-WT invasion (***p < 0.001). This further confirms the role of the *tolC* gene in the invasion process, as shown in Figure 1a. This suggests that the *tolC* gene is essential for *S*. Typhi’s ability to invade host epithelial cells.

(a) The invasion ability of *S*. Typhi strains was measured by infecting HT-29 and THP-1 cells and counting intracellular bacteria after gentamicin treatment. The data are presented as the mean ± standard deviation of three experiments performed in triplicate. The ST-Δ*tolC* showed significantly reduced invasion compared with the ST-WT strain (***P <0.001). (b) and (c) the expression of chemokines was assessed by infecting THP-1 macrophages with *S*. Typhi strains and measuring the expression of the *il-1β* and *il-8* genes by RT-PCR. The values are expressed as the mean ± standard error of the mean of three independent experiments. The ST-Δ*tolC* induced significantly more chemokine expression than the ST-WT strain (***p <0.001). Student’s t-test was performed to compare the values of the ST-∆*tolC* and ST-∆*tolC+* strains to the ST-WT strain. The values with a *p*-value of ≤ 0.001 are marked with an asterisk (***). This indicates a significant difference between the strains.

The intracellular presence of the ST-Δ*tolC* strain can be attributed to the phagocytic activity of macrophages. A distinct attenuation pattern was noted in the assays involving THP-1 cells. Through the phagocytic activity of macrophages, the ST-Δ*tolC* strain was able to enter the macrophages, although at a significantly lower rate than the ST-WT strain (29.00%, ***p< 0.001). Conversely, the ST-∆*tolC+* cells demonstrated a significantly higher invasion rate than the ST-WT strain (247.00%, ***p < 0.001). This suggests that the *tolC* gene might enhance the bacteria’s ability to resist the macrophages’ phagocytic activity, allowing them to invade more effectively. as shown in Figure 1a.

### *ST*-*Δ*tolC inside macrophages increases cytotoxicity and proinflammatory gene expression

TolC may either secrete an effector or directly interfere with innate immune pathways. Such interference could delay the activation of host cell death and diminish effective proinflammatory responses against intracellular pathogens. To evaluate this hypothesis, the *in vitro* expression of genes associated with proinflammatory chemokine (*il-8*) and cytotoxicity response (*il-1β*) was measured in THP-1 macrophages. These macrophages were infected with ST-WT, ST-Δ*tolC*, and ST-Δ*tolC+* strains of *S*. Typhi, alongside an uninfected negative control. The intracellular presence of ST-Δ*tolC* led to an increase in the expression of the *il-1β* chemokine in macrophages. Specifically, the ST-Δ*tolC* strain increased *il-1β* expression three-fold (*p* < 0.001) compared to the ST-WT strain, indicating cytotoxicity. However, the ST-Δ*tolC+* strain reduced *il-1β* expression below the ST-WT level at 2 hours post-infection (Figure 1b). Similarly, the intracellular presence of ST-Δ*tolC* triggered an increase in the expression of the proinflammatory chemokine *il-8* in macrophages. The ST-Δ*tolC* strain increased *il-8* expression twenty-fourfold (*p* < 0.001) compared to the ST-WT strain. However, the ST-Δ*tolC+* strain decreased *il-8* expression below the ST-WT level at 2 hours post-infection (Figure 1c). The increased expression of *IL-1β* and *IL-8* suggests that the deletion of the *tolC* gene in *S*. Typhi results in a more potent inflammatory response and cytotoxicity in the host cells. In contrast, the restoration of *tolC* in ST-Δ*tolC* led to a decrease in the expression of both *IL-1β* and *IL-8* below the levels of the ST-WT strain. This implies that the *tolC* gene might have a role in modulating the host’s immune response.

### Cytotoxic effect of intracellular *ST*-*Δ*tolC on macrophages

**Figure 2.**
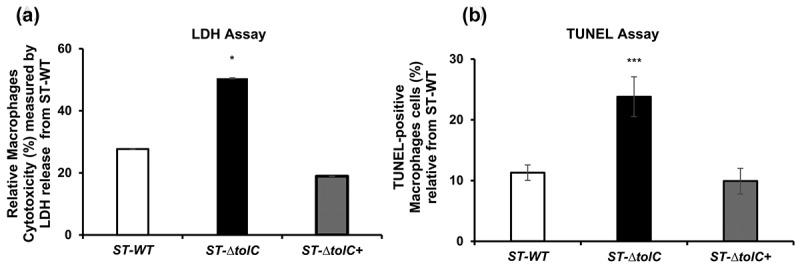
The ST-Δ*tolC* mutant induces cytotoxicity and pyroptosis in human THP-1 macrophages.

It was hypothesized that the attenuation of the ST-Δ*tolC* is due to the absence of a secreted toxin or a virulence factor. To assess this hypothesis, a comparative analysis was conducted between the toxicity levels of the ST-Δ*tolC* and the ST-WT strain within host cells. THP-1 macrophages were infected with either ST-Δ*tolC* or ST-WT at an MOI of 50, and lactate dehydrogenase release (LDH) was measured as a marker for cell death. LDH release was quantified at 4 hours post-infection. The results showed that ST-Δ*tolC* caused significantly more LDH release than the ST-WT strain (a 2–3-fold increase), as shown in Figure 2a. This indicates that the ST-Δ*tolC* mutant was more cytotoxic than the ST-WT strain. The complementation of ST-Δ*tolC* with a *tolC* expression plasmid reduced the toxicity of the mutant back to wild-type levels (Figure 2a). These results suggest that TolC is not only an exporter of cytotoxic factors, but possibly also exports protective factors for intracellular bacterial survival post-invasion.

The cell death and DNA fragmentation of THP-1 macrophages infected with the ST-WT, ST-Δ*tolC*, or ST-Δ*tolC*+ strain at an MOI of 50 were measured. (a) Cytotoxicity was quantified by measuring LDH release at 4 h post infection. The bars represent the mean ± SEM of three independent experiments. The ST-Δ*tolC* caused significantly more LDH release than the ST-WT strain (*P <0.05). (b) A pyroptosis was quantified by performing TUNEL staining at 4 h post infection. The percentages of TUNEL-positive cells ± SEM were calculated from 10 separate fields and represent the averages of three independent experiments. The ST-Δ*tolC* induced significantly more DNA fragmentation than the ST-WT strain (***P < 0.05). Student’s t-test was performed to compare the values of the ST-∆*tolC* and ST-∆*tolC+* strains to those of the ST-WT strain. The values with a *p*-value of ≤ 0.05 are marked with an asterisk (*). This indicates a significant difference between the strains.

The aim of this experiment was to explore whether the increased cytotoxicity of ST-Δ*tolC* was due to an elevated level of programmed cell death. TUNEL assays were performed on THP-1 macrophages infected with the ST-Δ*tolC* or ST-WT strain. TUNEL-positive cells were quantified using fluorescence microscopy. As shown in Figure 2b, the ST-Δ*tolC* induced more DNA fragmentation than the ST-WT strain. The number of TUNEL-positive cells was approximately 2-to 3-fold higher in macrophages infected with ST-Δ*tolC* compared to those infected with ST-WT. The complementation of the ST-Δ*tolC* with a *tolC* expression plasmid restored DNA fragmentation to wild-type levels. The increase in DNA fragmentation induced by the ST-Δ*tolC* correlated with the increase in cell death, as measured by LDH release. These results indicate that the ST-Δ*tolC* is defective in suppressing host cell death responses.

### Confirming the intracellular presence of *ST*-*Δ*tolC and cytotoxicity

**Figure 3.**
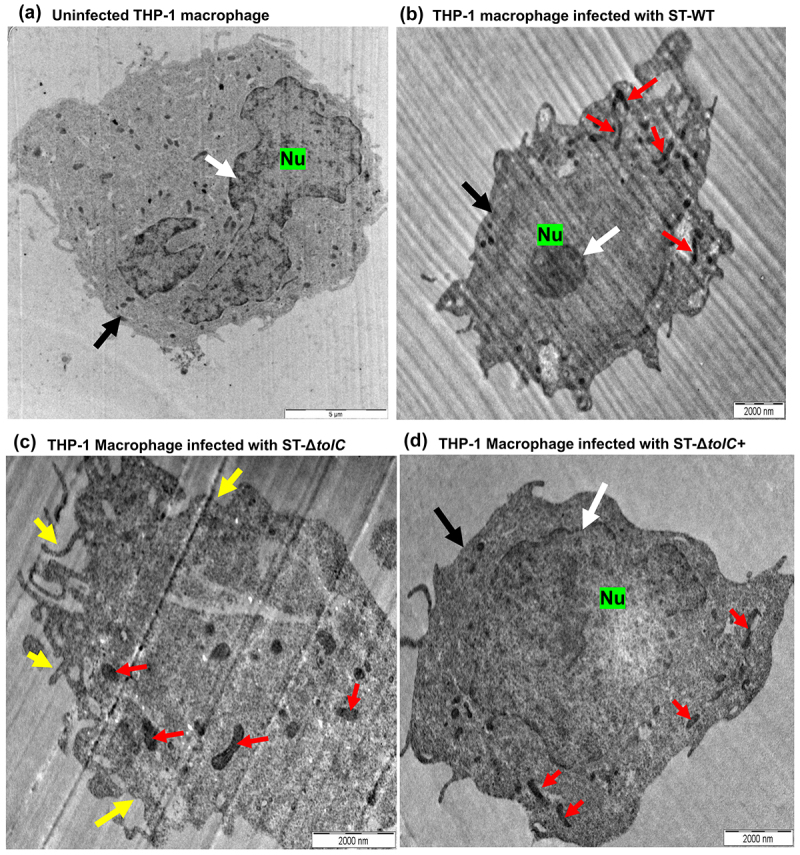
*S*. Typhi ST-Δ*tolC* was hypercytotoxic to THP-1-derived human macrophages.

The aim of this experiment was to determine whether the cytotoxicity of ST-∆*tolC* was observable when ST-∆*tolC* was inside the host cell. To test this hypothesis, two distinct cell lines, HT-29 and THP-1, were selected. THP-1 macrophages, which exhibit phagocytic activity during invasion assays, facilitate the entry of ST-Δ*tolC* into macrophages. In contrast, HT-29 cells do not possess phagocytic activity. This differential behaviour between the two cell lines provides a robust platform for assessing the intracellular behaviour and potential immune suppression abilities of ST-Δ*tolC*. Electron micrographs were utilized to examine the intracellular presence and cytotoxicity of *S*. Typhi strains on macrophages. Electron micrographs of macrophages infected with ST-Δ*tolC* revealed intracellular bacterial septa and phenotypic changes, such as the absence of a nucleus and defects in membrane integrity (Figure 3, c). These observations suggest the intracellular presence of ST-Δ*tolC*, which appears to cause more cytotoxicity in macrophages than the ST-WT or ST-Δ*tolC*+ strains. The introduction of the *tolC* expression plasmid reduced the cytotoxicity of ST-Δ*tolC*, reducing it similar to that of ST-WT (Figure 3, b, d).

In the TEM Analysis of THP-1-derived human macrophages: TEM images were captured 2 hours post-infection. The macrophages were infected with ST-WT, ST-Δ*tolC*, and ST-Δ*tolC*+ strains at a multiplicity of infection (MOI) of approximately 50.0. (a) Uninfected THP-1 macrophage: Throughout the 2-hour experiment, the macrophages remained healthy with an intact cell membrane. The cell displays a distinct nucleus (Nu), as indicated by the white arrow. The integrity of the macrophage membrane is highlighted by the black arrow. (b) THP-1 macrophage infected with ST-WT: Macrophages infected with ST-WT presented nuclear condensation and minimal plasma membrane damage after 2 hours post-infection. The replication of ST-WT bacilli was evident from the visible bacterial septa, as indicated by the red arrows. The dense nucleus (Nu) is highlighted by the white arrow, while the black arrow points to the intact membrane of the macrophages, indicating their integrity. (c) THP-1 Macrophage infected with ST-Δ*tolC*: Infection with the ST-Δ*tolC* strain results in phenotypic changes such as the absence of a nucleus and membrane integrity defects in macrophages. The image illustrates the absence of the nucleus (Nu). The yellow arrows point to the macrophages, highlighting their compromised membrane integrity. The bacterial septa are indicated by red arrows. (d) THP-1 Macrophage infected with ST-Δ*tolC*+: Macrophages infected with ST-Δ*tolC*+ demonstrated protection, as evidenced by the absence of cellular damage. The integrity of the host cell membrane was preserved, with no apparent damage. Compared to those infected with ST-WT, these macrophages appeared relatively healthier, with their cell membranes remaining intact. The large nucleus (Nu) is clearly visible, as highlighted by the white arrow. The black arrow points to the intact membrane of the macrophages, while the bacterial septa are indicated by red arrows.

**Figure 4.**
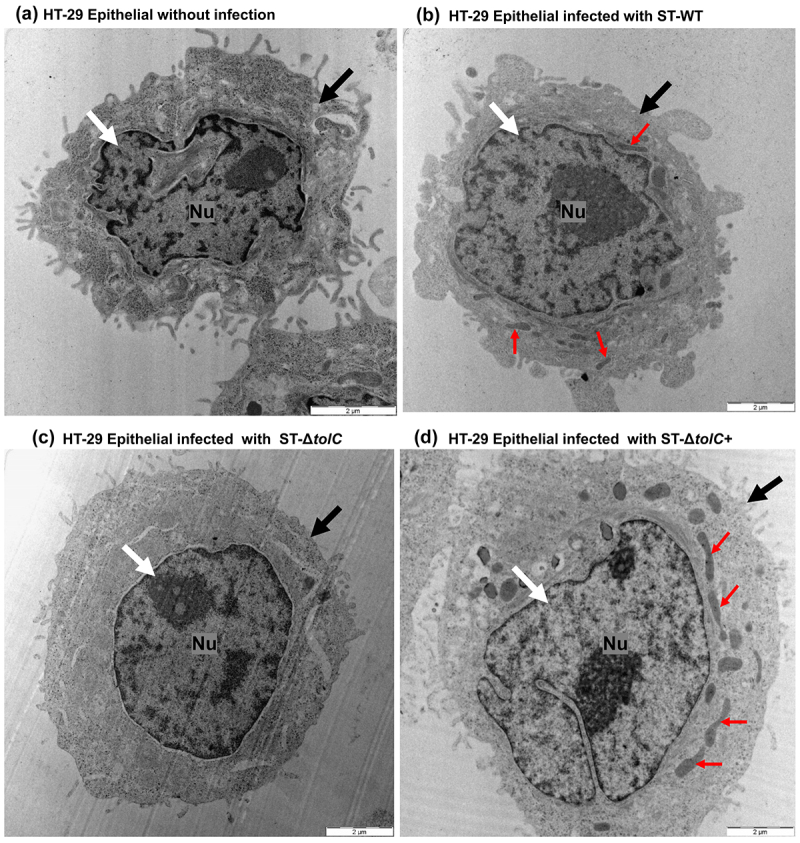
*S*. Typhi ST-Δ*tolC* was not hypercytotoxic to human gut epithelial HT-29 cells.In the TEM Analysis of human gut epithelial HT-29 cells: TEM images were acquired 2 hours post-infection. (a) Control HT-29: An uninfected HT-29 epithelial cell with a prominent nucleus (Nu) and intact cellular structure throughout the 2-hour experiment, the epithelial cell remained healthy with an intact cell membrane. The cell displays a distinct nucleus (Nu), as indicated by the white arrow. The integrity of the epithelial cell membrane is highlighted by the black arrow. (b) HT-29 cell infected with ST-WT: HT-29 cells presented clear nuclear and minimal plasma membrane damage after 2 hours post-infection. The replication of ST-WT bacilli was evident from the visible bacterial septa, as indicated by the red arrows. The dense nucleus (Nu) is highlighted by the white arrow, while the black arrow points to the intact membrane of the HT-29 cells. (c) HT-29 cell infected with ST-Δ*tolC*: HT-29 cell showing relatively preserved cellular architecture with absence of any alterations. White arrows indicate the nucleus and black arrows indicate the intact membrane of epithelial cells. Significantly, the lack of ST-Δ*tolC* bacilli inside the HT-29 cells was apparent due to the complete absence of bacterial septa. This demonstrates that ST-Δ*tolC* was unsuccessful in invading the HT-29 cells. (d) HT-29 cell infected with ST-Δ*tolC+*: The HT-29 cell shown no significant cellular damage after infection. The cell membrane remained intact, showing no visible damage. The large nucleus (Nu) is easily identifiable, as pointed out by the white arrow. The black arrow indicates the undamaged membrane of the macrophages, while the presence of bacterial septa is marked by red arrows.

To further determine whether the cytotoxicity of ST-Δ*tolC* on macrophages was due to its intracellular presence, electron micrographs of the HT-29 cell infected with ST-Δ*tolC* were examined. The cellular architecture of the HT-29 cell remained relatively intact, showing no significant alterations. Notably, the absence of ST-Δ*tolC* bacilli within the HT-29 cells was evident, as there were no bacterial septa present (Figure 4c). This observation underscores the fact that ST-Δ*tolC* was unable to invade the HT-29 cells. However, ST-Δ*tolC* was found within macrophages, a result of the phagocytic activity of these cells. Electron micrographs of HT-29 cells infected with other strains did not show cytotoxicity and had intact nuclei and membranes, regardless of the strain (Figure 4b,d). This suggests that the *tolC* gene might play a role in modulating the cytotoxic effects of *S*. Typhi on host cells.

### Structural integrity defects on *ST*-*∆*tolC cells

**Figure 5.**
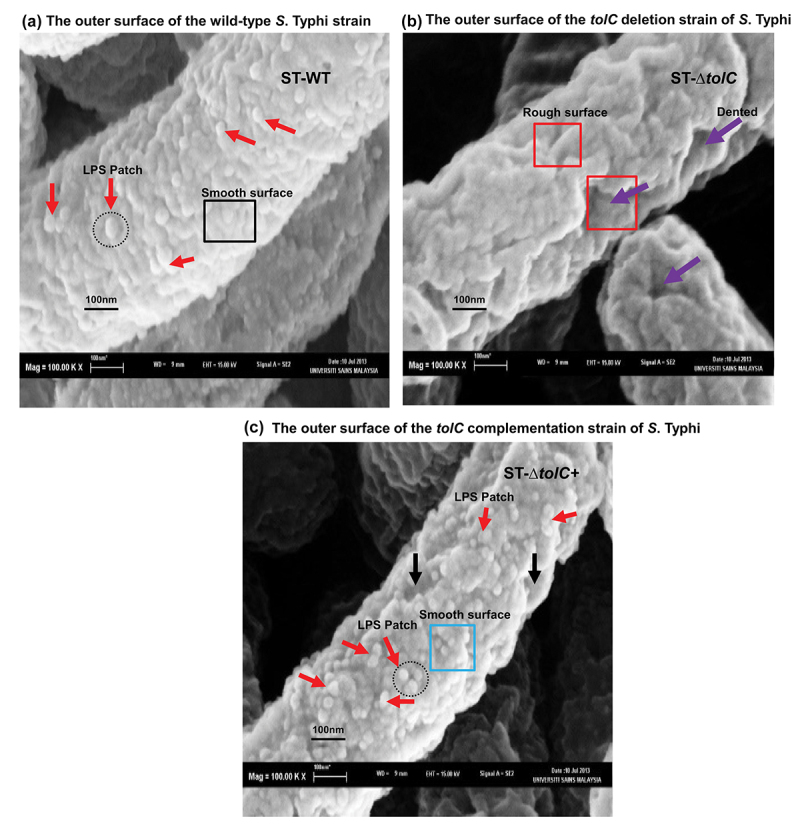
The deletion of *tolC* decreases the integrity of the outer membrane, affecting the morphology of the cell surface. (a – c) Scanning electron micrographs reveal distinct changes in the cell surface appearance of the ST-Δ*tolC*, demonstrating phenotypic heterogeneity in comparison to the ST-WT and ST-Δ*tolC*+ strains. (a) The ST-WT strain, highlighted by the black square, shows a smooth surface. The presence of LPS patches is indicated by red arrows and a black dotted circle. (b) In contrast, the ST-Δ*tolC* strain, marked by red squares, displays a rough surface lacking visible LPS patches. Numerous dented areas are indicated by purple arrows. (c) The ST-∆*tolC*+ strain, denoted by the blue square, presents a smoother surface with LPS patches, as indicated by red arrows and a black dotted circle. This strain shows fewer dented areas, pointed out by black arrows. It’s important to note that the complementation of ST-Δ*tolC* did not completely reverse the effects of the *tolC* deletion. All cells were observed at a magnification of 100,000X, with scale bars representing 100 nm.

Defects in the bacterial membrane could induce hypercytotoxicity in host cells. To test this hypothesis, SEM was employed to compare the cell surfaces of *S*. Typhi strains: ST-WT, ST-∆*tolC*, and ST-Δ*tolC*+. The ST-WT strain presented a smooth surface with visible lipopolysaccharide patches (LPS patches), as shown in Figure 5a. In contrast, the ST-Δ*tolC* strain revealed a rough, dented surface with no visible LPS patches (Figure 5b). The smoother surface with LPS patches displayed by the ST-∆*tolC*+ strain, compared to the ST-∆*tolC* strain (Figure 5c), indicates that the complementation reversed the effects of the *tolC* deletion. These differences were consistent across all cells of each strain (Figure 5a-c). This suggests that the *tolC* gene plays a significant role in maintaining the outer surface structure of *S*. Typhi, and the surface quality of the bacterial strains could be linked to their cytotoxic effects on host cells.

## Discussion

TolC has been shown to facilitate inhibitory function that is effective against cell death pathways, and the activation of specific cell death pathways is dependent on certain infection conditions and type of host cells [14]. *S*. Typhi can infect the host without triggering a noticeable inflammatory response by upregulating the Vi capsule, which conceals its LPS and flagellin [5,6]. These molecules are normally recognized by TLR-4 and TLR-5 on host cells, leading to the production of IL-8, a neutrophil chemoattractant [33]. However, in the absence of IL-8, no neutrophil recruitment or localized inflammation occurs at this stage of infection [1–3,34]. This may also explain why *S*. Typhi-infected patients do not experience diarrhoea because IL-8 is involved in intestinal fluid secretion. Therefore, it is hypothesized that *S*. Typhi has unique virulence factors or mechanisms that allow it to suppress the innate immune response in the intestinal mucosa, assisting its systemic dissemination [2,3,24]. The ability of *S*. Typhi to suppress and delay host immune responses during infection most likely enables the bacteria to benefit from the host and cause disease. However, the precise molecular mechanisms of the function(s) of TolC in the immunomodulatory ability of *S*. Typhi remains unknown.

The hypothesis is that TolC plays a dual role – it either facilitates the secretion of an effector or directly disrupts innate immune pathways. This disruption could potentially delay the initiation of host cell death, thereby diminishing the effectiveness of proinflammatory responses against intracellular pathogens. To evaluate the validity of this hypothesis, The invasion ability and *in vitro* expression of genes related to the proinflammatory chemokine (*il-8*) and cytotoxicity response (*il-1β*) were measured in human macrophages infected with ST-WT, ST-Δ*tolC*, and ST-Δ*tolC*+ strains of *S*. Typhi, along with an uninfected negative control (Figure 1a-c). Cell cytotoxicity assays (Figure 2a,b) and transmission electron microscopy (TEM) analysis of the infected epithelium and macrophages were performed to see the cytotoxic effect of *S*. Typhi strains on infected cells, with an uninfected negative control (Figures 3 and 4). The cytotoxic effect may cause damage to host cells, such as cell integrity defects and fragmented cells without a nucleus, and these defects are included as markers for host cell death [14]. Moreover, we also observed outer membrane integrity defects in ST-Δt*olC (*Figure 5b)

TolC may affect the virulence of *S*. Typhi. Thus, the *tolC* mutant of *S*. Typhi had a reduced ability to invade human epithelial and macrophages in a cell culture infection model. However, we recovered a small fraction of ST-Δ*tolC* from macrophages, but not from epithelial cells, as macrophages had phagocytic activity during the invasion assay. The presence of ST-Δ*tolC* inside the macrophages could have an impact on host cell pathways compared with the ST-WT strain, as shown in Figure 1a. The intracellular presence of ST-Δ*tolC* could potentially elucidate the function of the *tolC* gene within the host cell.

when the *tolC* gene was restored in the ST-Δ*tolC* strain, the bacteria were able to invade the macrophages even more than the wild-type strain (247.00% of the invasion rate of the wild-type strain) as shown in Figure 1a. This suggests that the *tolC* gene might help *S*. Typhi to evade the immune response by resisting the phagocytic activity of macrophages, thereby enhancing its ability to invade host cells and establish an infection. This could be a potential mechanism that *S*. Typhi uses to cause disease in its host. Further research is essential, to validate this hypothesis and gain a comprehensive understanding of the underlying mechanisms.

The deletion of the *tolC* gene in *S*. Typhi led to an increased expression of proinflammatory chemokines (*IL-8*) and cytotoxicity response genes (*IL-1β*) in infected macrophages (Figure 1b,c). This suggests that the *tolC* might normally act to suppress these responses, possibly by interfering with the signalling pathways that lead to their activation. Understanding these mechanisms could potentially help in the development of new treatments or vaccines for typhoid fever.

Further study is needed to determine whether TolC or its released factor (s) can suppress host immune responses from outside of host cells. However, we found that when the ST-∆*tolC* was present outside of the HT-29 cells, it did not cause cytotoxicity in HT-29 cells. This suggests that TolC may be more effective at suppressing the host immune response when it is located within the host cells. Therefore, both the ST-WT and ST-∆*tolC+* strains suppressed the host cell response by invading and staying inside the cells (Figure 1a-c, Figure 2 a,b, Figure 3 b,d, and Figure 4b,d). The ST-∆*tolC* had a cytotoxic effect on host macrophages (Figure 3c). This might be because ST-∆*tolC* was inside macrophages, which have phagocytic activity (Figure 1a). On the other hand, HT-29 cells did not show cytotoxic effects after infection with any strains (Figure 4b-d). This is because the HT-29 epithelium is not phagocytic, so the ST-∆*tolC* could not enter these cells (Figure 1a, Figure 4c). This supports the hypothesis that the ST-∆*tolC* inside the host cells causes cytotoxic effects and may induce pyroptosis by activating caspase-1 in infected human macrophages. Moreover, this increased cytotoxicity may be related to caspase-1 activation. According to this, increased expression of a cytotoxicity marker (*il-1ß*) was observed in the ST-∆*tolC*-infected macrophages (Figure 1b). Our results show that TolC plays a role in *S*. Typhi’s ability to modulate the immune system. These results suggest that the ST-∆*tolC* was attenuated not only because of its hypercytotoxicity and proinflammatory response activation but also because of the early loss of its intracellular niche.

Intracellular pathogens use host cell death suppression to prolong their reproduction in the host [35]. Bacteria use various mechanisms to avoid apoptosis, such as activating host cell survival pathways, blocking cytochrome c release by mitochondria, and inhibiting caspase activity [35]. Some intracellular pathogens such as *Legionella* and *Shigella*, deliver effector proteins to host cells through secretion systems to prevent apoptosis [36–38]. Other pathogens, such as *Wolbachia* and *Neisseria*, use their surface proteins to stop host cell apoptosis [39,40]. Our results concur with the *F. tularensis* study [14] which showed that *tolC* mutant caused a significant increase in the proinflammatory chemokine, IL-8. Our results are also in agreement with another study that showed that the *tolC* mutant of *F. tularensis* suppressed intrinsic apoptotic pathway activation in a TolC-dependent manner during primary macrophage infection and mouse organ colonization [41].

Using SEM, the surface structure of *S*. Typhi strains ST-WT, ST-Δ*tolC*, and ST-Δ*tolC*+ was visualized (Figure 5). The surfaces of ST-WT and ST-Δ*tolC*+ showed numerous protrusions or bumps, which can be identified as LPS aggregations on the bacterial membrane [42–44]. These aggregations, referred to as LPS patches as shown in Figure 5. These LPS patches, often in groups of 600 to 3500 molecules, cover a significant portion of the cell surface [44].

Several differences between ST-WT and ST-Δ*tolC* surface structures were observed. The ST-WT strain presented a smooth surface with visible LPS patches. In contrast, the ST-Δ*tolC* strain revealed a rough, dented surface with no visible LPS patches (Figure 5b). The smoother surface with LPS patches displayed by the ST-∆*tolC*+ strain, compared to the ST-∆*tolC* strain (Figure 5c), indicates that the complementation reversed the effects of the *tolC* deletion. In our study, LPS patches were more clearly visible at the 100 nm scale bars, representing magnified views of the outer membrane, compared to the previous study [42]. Scanning electron micrographs at the scale bars 100 nm represent the magnified views that highlight the features that reveal distinct changes in the cell surface appearance of the ST-Δ*tolC*, demonstrating phenotypic heterogeneity in comparison to the ST-WT and ST-Δ*tolC*+ strains. These results suggest the *tolC* gene appears to play a significant role in maintaining the outer surface structure of *S*. Typhi.

The presence of LPS on the bacterial surface contributes to the virulence of the bacteria and the host’s inflammatory response during infection [45]. Some bacteria can modify the structure of LPS to evade the host’s innate immunity [46,47]. For example, changes in the acylation pattern of lipid A, a component of LPS, can reduce its recognition by the Toll-like receptor 4 (TLR4)/MD-2 complex, a key player in the innate immune response. This can lead to a weaker immune response, facilitating bacterial evasion [48]. The LPS patches observed in the different strains of *S*. Typhi could be indicative of these functions and their potential implications for bacterial pathogenicity or antibiotic resistance.

Another possible interpretation, the *tolC* mutant may induce hypercytotoxicity in host cells. This might be because the damaged *S*. Typhi cell membrane (Figure 5b) released bacterial DNA which triggered caspase-1 pyroptosis instead of apoptosis. Defects in the bacterial membrane could induce hypercytotoxicity in host cells [19–23]. In contrast, TolC-facilitated suppression of apoptosis is an active process and may not be caused by defects in the structural integrity of the *tolC* mutant [41].

TolC, which is present in the bacterial outer membrane, may interact with host proteins to stop host cell death pathways. For instance, *Wolbachia* and *Neisseria* use their surface proteins to prevent cell death pathways [39,40]. However, the more established view is that TolC is needed for toxin secretion and plays a key role in the type I secretion system. A previous report showed that a *tolC* mutant of *F. tularensis* subspecies *novicida* U112 could not release functional hemolysin [49]. Haemolysin is a common substrate of the type I secretion system and, can affect cell death pathway signalling proteins. We note that our HT-29 cell experiments were similar to a previous study that used HUVEC cells [14]. HT-29 cells and HUVEC cells are alike because they are not phagocytes and do not take up pathogens [50]. Based on our results, TolC or its released factor or factors directly influenced the ability of *S*. Typhi to inhibit proinflammatory responses and the cell death pathway of the host to an extent.

In our study, we emphasized the potential of TolC as a target for novel therapeutics against typhoid fever. This assertion is based on the unique role of TolC in bacterial physiology and pathogenesis. TolC presents a promising target for antimicrobial intervention. Recent studies have explored this potential. For instance, research on novel antimicrobial agents has identified compounds that can inhibit TolC function, thereby sensitizing bacteria to existing antibiotics. This approach could help overcome the challenge of antibiotic resistance, a growing concern in the treatment of typhoid fever and other bacterial infections [51,52]. The advent of the in-silico approach has revolutionized vaccine design. By using computational tools, researchers can identify potential vaccine targets like TolC more efficiently. These tools can predict antigenic properties and stimulate immune responses, thus accelerating the vaccine development process [53]. Recently, a novel approach has been investigated to discover inhibitors of AcrAB−TolC, including disruption of the pump assembly using specially designed peptides, which can mimic the transmembrane helices of AcrB and can disrupt the trimerization of AcrB. This disruption results in the inhibition of drug efflux facilitated by AcrAB−TolC [54]. While our study did not examine these aspects, the potential of TolC as a target for novel therapeutics against typhoid fever is evident. Future research should continue to explore this promising avenue.

In conclusion, it was found that TolC functioned in the virulence of *S*. Typhi. Therefore, the ST-∆*tolC* failed to suppress the host cell death response and induced a proinflammatory immune response from macrophages. It is possible that TolC or its released effectors directly interrupt the protective pathways of host cells. This interruption might give more time for the bacteria to replicate in a safe intracellular environment. TolC-dependent host-suppressive abilities may be important for the severe virulence of *S*. Typhi in the human host. Based on current information, this is the first report that expands the phenotypes related to *tolC* deletion in *S*. Typhi. Our findings suggest that the intracellular presence of the *tolC* mutant, which has a defective cell membrane, could potentially cause cytotoxicity in human macrophages. This study could also validate results from previous reports that have shown TolC plays an important role in the pathogenesis and virulence of *Escherichia coli* and *Enterobacteriaceae*. If the *tolC* gene is indeed responsible for maintaining the bacterial surface structure and modulating its cytotoxic effects, it could be a potential target for new drugs or therapies.

## Data Availability

The data that support the findings of this study are openly available in 10.6084/m9.figshare.26130901
